# Determinants of Non-Adherence to Anti-Tuberculosis Treatment in a Public Primary Healthcare Clinic in South Africa: Improving the Quality of Long-Term Care

**DOI:** 10.3390/ijerph22081209

**Published:** 2025-07-31

**Authors:** Lucky Norah Katende-Kyenda

**Affiliations:** Department of Internal Medicine and Pharmacology, Faculty of Medicine and Health Sciences, Walter Sisulu University, Sissons Street, Fortgale, Mthatha 5117, Eastern Cape, South Africa; nkatende-kyenda@wsu.ac.za; Tel.: +27-631563969

**Keywords:** South Africa, non-adherence, tuberculosis, primary healthcare, direct observed treatments, public health

## Abstract

Background: Non-adherence to anti-tuberculosis treatment remains a major obstacle to increasing tuberculosis treatment success rates and enhancing healthcare expenditure. The aim of this study was to identify determinants contributing to non-adherence to anti-tuberculosis treatment in a public primary healthcare clinic in South Africa. Method: A cross-sectional study was carried out to collect data from 65 participants using face-to-face interviews with a structured questionnaire. Data were analyzed using SPSS. Results: Of the 65 participants interviewed, 41 (63.08%) were males and 24 (36.92%) were females. A total of 45 (69.23%) were adherents and 20 (30.77%) were non-adherents. Gender was the major predictor of non-adherence with more males committed to treatment than females with a significant association (X^2^ = 65.00 and *p* of <0.001). Conclusions: The major contributing factors to non-adherence were long dis-tances to the clinics, a lack of family support, and unemployment. Comprehensive programs addressing these multifactorial factors are needed for successful treatment and eradication of tuberculosis.

## 1. Introduction

Tuberculosis (TB) is an infectious disease that remains the leading cause of death among adults worldwide [1]. Tuberculosis is a preventable and curable disease, yet it likely reclaimed its position as the deadliest infectious disease in the world, surpassing COVID-19 [2]. Tuberculosis causes not only direct health consequences, but also significant social and economic burdens, including healthcare costs, lost wages, and psychological distress. TB is responsible for more than 1 million deaths annually, which has devastating impacts on families and communities [3]. It causes almost twice as many deaths as Human immunodefiency virus/Acquired immunodefiency syndrome (HIV/AIDS) and impacts more than 10 million individuals each year, with case numbers increasing since 2021 [1]. Urgent action is required to achieve the United Nations (UN) and WHO target of ending the global TB epidemic by 2030.

In 2023 alone, TB was responsible for 1.25 million deaths, including 161,000 among people living with HIV. While 1.5 million people succumb to TB annually, over 10 million new cases emerge every year [4]. The burden of TB is disproportionately borne by developing countries, where 75% of cases occur among the most economically productive age group (15–54 years), exacerbating the socioeconomic impact of the disease [1].

The WHO aims for a cure rate of at least 85% for all diagnosed TB cases. Therefore, adherence to treatment needs to be between 85–90%, especially for those people on anti-retroviral therapy (ART) [5]. Global efforts to combat TB have been successful, with a Millennium Development Goal (MDG) target to halt and reverse TB incidence by 2015. This will lead to a significant reduction in TB mortality rates, saving an estimated 43 million lives between 2000 and 2014 [6]. However, some countries, like South Africa (SA), still struggle with low cure rates, with only about 54% of TB patients being cured—far below the target of the WHO [7].

In 2023, approximately 10.8 million people worldwide contracted TB, including 6 million men, 3.5 million women, and 1.3 million children [4]. What is concerning is that TB is present in all countries and age groups, and multidrug-resistant TB (MDR-TB) remains a significant public health crisis. Nevertheless, it is important to emphasize that TB is both preventable and curable. The WHO in 2025 reported that the 2030 targets represent a 90% reduction in TB deaths and 80% reduction in TB incidence compared with 2015 levels [1]. The 2035 targets are for reductions of 95% and 90%, respectively [1]. A third target that no TB-affected households should experience catastrophic costs due to the disease by 2020 was also agreed [8]. By 2030, the target is to achieve a 90% reduction in TB-related deaths and an 80% decrease in the TB incidence rate compared to 2015 levels [9].

It was highlighted that an annual investment of USD 22 billion is essential for the effective prevention, diagnosis, treatment and care of TB [10]. This level of funding is critical to meeting the global TB targets by 2027, as established during the 2023 UN high-level meeting on TB. The End TB Strategy also sets out clear milestones and objectives aimed at reducing TB incidence and mortality. To address TB, specific milestones were established for 2020, including a 35% reduction in TB-related deaths, a 20% decrease in the TB incidence rate, and elimination of catastrophic costs for TB patients and their families. Looking forward, ending the TB epidemic by 2030 remains a crucial health objective under the Sustainable Development Goals (SDGs) of the United Nations [11].

In 2014, the global TB burden remained high, with 9.6 million new cases and 1.5 million deaths reported. While the specific number of countries can vary based on the year and criteria used by the WHO, the statement that twenty-two countries account for 80% of all TB cases is generally accurate [1]. It is considered that Direct Outcome Treatment [DOT] adaptability, through hybrid models combining digital tools and community engagement, has proven to be cost-effective and patient-centered, thus enhancing adherence, especially in high-risk groups [12]. The 2018 Global TB Report emphasized the success of the DOTS strategy, which was shown to enhance treatment adherence, lower recurrence rates, and prevent the development of MDR-TB [13]. Introduced by the WHO in 1995, DOTS comprises five essential elements: case detection through sputum smear microscopy and rapid molecular diagnostics, along with standardized treatment supported by supervision and patient care [13].

Non-adherence to ATT (Anti-Tuberculosis Treatment) has far-reaching consequences, leading to prolonged and severe illness, increased risk of death, disease transmission, and drug resistance [14]. These outcomes not only devastate individuals but also impose a substantial economic burden on both patients and the healthcare system. A study carried out in Indonesia also emphasized that non-adherence to TB treatment poses a major challenge to global TB control, significantly leading to treatment failures and impeding effective disease management [15].

A systematic review carried out established that the factors that were most frequently consistently and statistically significantly related to non-adherence to TB treatment were as follows: family income, patient movement and changing the address or giving the wrong address, and TB relapses or multidrug-resistant TB (MDR-TB) during the intensive phase of treatment [16]. Other factors include a history of default, the treatment regimen (long course), response to treatment, homelessness, stigma, seeking help from traditional healers, staff receptiveness, lack of DOTS, poor knowledge or lack of health education, side-effects of drugs, feeling better, alcohol intake, and a lack of family and social support.

As the prototypical disease of poverty, it is considered that undernutrition and overcrowding remain the major social determinants of TB in a high-burden country such as SA. Compounding these drivers are other biosocial risk factors, namely, HIV co-infection, alcohol use disorders, smoking, and diabetes [17].

A study carried out on the economic impact of TB mortality revealed that more than 90% of global TB cases and deaths occur in the developing world, where approximately three-quarters of these cases affect the most economically productive age group (15–54 years), with significant socioeconomic impacts [18]. This is because TB is primarily a disease of adults in their most productive years. The high incidence of TB in this age group significantly impacts families and communities due to lost income, healthcare costs, and other economic burdens.

To control transmission and prevent medication resistance, WHO [19] recommends that at least 85% of all identified TB cases be cured, with a focus on obtaining high cure rates among smear-positive pulmonary TB patients. South Africa has a particularly high burden of TB, with an incidence rate of 468 per 100,000 of the population. In 2022, health authorities recorded an estimated 54,000 deaths from TB. However, with support from the WHO and partners, the country has become a trailblazer in its bid to tackle this epidemic [19].

Based on the total number of TB cases, SA was at number four on the list of countries with a high TB burden in 2018 [20]. Results from a study carried out on inequities revealed that social, economic, and clinical barriers to care continue to impede TB control efforts [21]. Poor adherence to recommended anti-TB treatment programs, such as those covered by the DOTS strategy, results from these. In the end, this is one of the reasons why TB cure rates are so low.

Despite the introduction of the DOTS program in 1995, it was noted that TB continues to be a major health challenge in SA, especially in the Eastern Cape (EC) [22]. Although TB incidence has been declining in SA since 2009 and deaths due to TB have decreased in recent years, it is undisputed that TB remains the number one cause of death in this country [23]. The Global TB Report 2015 estimated that in 2014, SA had the second-highest TB incidence rate in the world, with 834 cases per 100,000 population.

Studies have indicated that perceptions, rather than mere knowledge, heavily influence TB treatment initiation and non-adherence in this region. While the WHO recommends a TB cure rate of 85%, the EC reports a much lower cure rate of 41%, falling short of both the national average and the minimum recommended threshold of 65% of the WHO [1]. The EC province of SA faces a high burden of TB, characterized by elevated incidence and prevalence rates alongside notably low cure rates [7].

It is important to gain knowledge about TB disease, prevention, treatment and sources of information, attitudes towards TB patients and their stigmatization, and diagnosis and treatment practices in the general population of middle- and low-income countries with a high TB burden to provide evidence for policy development and decision making [24].

Healthcare workers (HCWs) generally demonstrate good knowledge of TB, but gaps remain, particularly regarding MDR-TB and XDR-TB. Attitudes towards patients with TB and infection control practices are often less than optimal, even among those with good knowledge. This discrepancy highlights the need for targeted interventions to improve both knowledge and practices among HCWs [25].

Tuberculosis is the second-deadliest infectious disease in the world. Despite the availability of drugs to cure TB, control of TB is hampered by the emergence of MDR-TB and XDR-TB. The presence of MDR/XDR-TB is alarming due to the low detection rate, high treatment failure, and high mortality. MDR/XDR-TB presents significant challenges in diagnosis, treatment, and prevention, while also creating opportunities for innovation and improved strategies. Key challenges include limited diagnostic tools, lengthy and toxic treatment regimens, high treatment failure and mortality rates, and the potential for further drug resistance development [26].

Investigating the determinants of non-adherence to ATT in a public primary healthcare (PHC) setting in SA has significant scientific value. It can enable identification of the key factors contributing to treatment failure and drug resistance, informing targeted interventions to improve adherence and treatment outcomes. Furthermore, the present research can also contribute to a broader understanding of the social and health system factors influencing TB control efforts in the South African context.

Adherence to TB medication is essential to the success of TB treatment. Suboptimal medication adherence is widespread in diseases that require long-term therapy, such as diabetes, hypertension, bronchial asthma, and TB [27]. Medication adherence is a complex human behavior that can be influenced by various factors, including patient-related health system, condition-related, therapy-related, and socioeconomic factors. The WHO reports the prevalence of TB annually in its Global TB Report. These data are crucial for several scientific reasons, including understanding the global TB epidemic, tracking progress towards TB elimination goals, and informing public health interventions.

### 1.1. Conceptual Framework

To understand further factors contributing to non-adherence, it is necessary to consider the theoretical frameworks that underpin why people do not adhere to TB treatment. There are two theoretical frameworks applied in the current study, namely, the Health Belief Model (HBM) and the World Health Organization Multidimensional Adherence Model (WHO-MAM). According to WHO-HBM, people’s beliefs about the severity of the disease, their susceptibility to it, the benefits of treatment, and the barriers to treatment, all influence their adherence. Based on HBM theoretical concepts, the current study applies the model to determine the effect of associated factors and individual patient’s perceptions of TB treatment adherence [28]. Understanding the level of adherence and how it relates to perception about its treatment provides an opportunity to design interventions that may improve adherence through overcoming misperceptions.

The World Health Organization - Multidimensional Adherence Model (WHO-MAM) [29] is a psychological model that recognizes that adherence is influenced by a combination of factors, including patient-related factors (like beliefs and knowledge), social and economic factors, and health system factors. It recognizes that these factors are interconnected with and influence each other [30].

The two theories are connected and inform the stated problem of this study that non-adherence to TB treatment among TB patients caused by multiple factors remains one of the primary challenges hindering improved cure rates. Non-adherence to TB treatment threatens the success of treatment, increases the risk of TB spreading, causes drug resistance, and increases morbidity and mortality.

### 1.2. Significance of This Study

This study highlights that non-adherence to TB treatment is associated with the socio-demographic characteristics of TB patients. This results in treatment failure, the development of MDR-TB, prolonged infectiousness, and overall poor treatment outcomes [14]. Additionally, in individuals with HIV, untreated TB infection can accelerate the progression of HIV as a disease. TB can worsen the ability of the immune system to fight HIV, leading to a faster decline in CD4 cell count and an increased viral load, ultimately accelerating the progression to AIDS. In patients with HIV infection, untreated TB infection accelerates HIV development [31]. Therefore, it is necessary to monitor the non-adherence of TB patients who are taking TB treatment. Comprehensive programs addressing these multiple factors are needed for the successful treatment and eradication of TB. In this way optimum health outcomes will be achieved. In response to this concern, the present study aimed to assess the determinants of non-adherence to anti-TB therapy in TB patients.

### 1.3. Aim and Objectives

The study aimed to identify the determinants contributing to non-adherence to anti-TB treatment among patients attending a TB clinic at a public primary healthcare facility in South Africa in 2023. This aim was achieved through the following specific objectives which were to:

- Describe the demographics of TB patients on TB treatment.
- Determine the prevalence of non-adherence of TB treatment.
- Identify determinants contributing to non-adherence of TB treatments.
- Identify the demographic characteristics associated with non-adherence and the determinants of non-adherence to ATT.
- Describe the patient programs and drug-related factors that affect non-adherence to ATT.

## 2. Materials and Methods

### 2.1. Study Design

A descriptive cross-sectional research approach was used in this study to evaluate the main determinants of non-adherence to tuberculosis treatment. The design was used by the researchers to analyze various factors, including demographic information, behaviors, conditions, and outcomes, to discern patterns or correlations within the population studied.

### 2.2. Setting

The study was conducted at Mthatha Gateway Clinic, a public primary health care clinic, in the Eastern Cape of South Africa. This healthcare facility was the only one selected to test the methodology so that the results could be compared with other healthcare facilities in future. Furthermore, the facility caters for a wide population. The clinic caters for the following local communities: Southridge, Fortgale, Lindale, Qweqwe, Ultra City, Slovo, Mandela Park, Chris Hani, Payne and Zimbane.

### 2.3. Study Population

The study population was TB patients attending the public primary healthcare clinic who were receiving anti-TB treatment from the clinic.

*The inclusion criteria were as follows:*

- Adults who were at least 18 years and older.
- Individuals who had been diagnosed with TB and had commenced TB treatment.
- Participants who voluntarily agreed to participate in the study.

*The exclusion criteria were as follows*:

- Participants with other active infections, such as MDR TB or XDR TB.
- Participants with severe cognitive impairments affecting their understanding.

#### 2.3.1. *Sample Size Calculation and Sampling Strategy*

The sample size was calculated using Slovin’s formula (Slovin, 1960) [32] n = N/(1 + Ne^2^), where n = sample size, N = population size, and e = margin of error. Given a population size of 280, the number of patients seen per day, and a margin of error <10% = 0.09, the sample size was calculated as follows:

n = N/(1 + Ne^2^)

n = 280/(1 + 280 × (0.09)^2^)

n = 280/(1 + 280 × 0.0081)

n = 280/(1 + 2.268)

n = 280/3.268

n = 85.679

n = 86

Therefore, our sample size was indicated to be 86 participants.

Due to those not wanting to participate, and some participants not being available, the number of participants interviewed was 65. This was the number used for data analysis.

#### 2.3.2. *Sampling Strategy*

A random sampling strategy was used to select patients. It is most appropriate for investigating non-adherence to TB treatment, particularly when seeking to understand the diverse experiences and factors contributing to this issue. This method allows the researchers to intentionally select participants who can provide rich insights into the problem based on specific characteristics like socio-demographic background, remoteness, or experiences with TB care.

### 2.4. Data Collection Process

#### 2.4.1. Data Collection Tool

A standardized questionnaire was used as a data collection tool. The questionnaire method is a versatile and potent tool used for data collection across diverse research domains. Its structured format facilitates standardized data collection, organization, and analysis, and is particularly advantageous for quantitative research endeavors [33]. The use of structured questionnaires aims to ensure both validity and reliability. Validity ensures the questionnaire measures what it is intended to do, while reliability guarantees consistent results when administered repeatedly. Reliability is generally considered high in structured questionnaires due to the use of standardized, closed-ended questions. Data were collected over a period of one week. This was done because the targeted patients were only available for that week when they came for their reviews by doctors.

#### 2.4.2. Variables

Demographic variables such as gender, age, marital status, educational status, income status, and HIV status were recorded. These were coded as either binary or specific: gender (male/female), age (18–25, 26–35, 36–45, 46–55 and >55), marital status (single/married), educational status (high school/tertiary), income status (ZAR) (unemployed, 1000–3000, 3001–5000, >5000), and HIV status (sero-positive/sero-negative). Section II consisted of factors contributing to non-adherence of TB treatment, including the patient program (substance use, distance from the clinic, interaction with the staff clinic, poverty) and drug-related factors (adverse effects of the treatment, shortage of drugs at the clinic). A non-adherent to tuberculosis drugs was defined as a patient who missed at least one scheduled dose of their tuberculosis medication. To accommodate the language barrier, the questionnaire, participant information sheet, and consent form were translated into IsiXhosa, a local language of the participants.

#### 2.4.3. Data Management

Data management involves the processes of collecting, organizing, safeguarding, and storing the data of an organization to enable effective analysis and interpretation. In this study, these activities were carried out as follows.

##### Data Cleaning Procedures 

The data cleaning procedures undertaken were as follows: Duplicates were identified and removed to ensure accuracy and avoid skewed results. Thereafter, the incomplete questionnaires were handled by deleting rows to ensure that the data were formatted consistently to avoid errors during analysis. Data entry errors were identified and fixed. Outliers were identified, and accuracy was verified for the cleaned data. There were 21 individuals that refused to participate. Therefore, the data analyzed were for 65 participants.

##### Data Protection

The data protection and confidentiality protocols included aspects to address theft, loss, and tampering of data.

#### 2.4.4. Data Analysis

Data cleaning: All questionnaires were checked for completeness. The coding was performed and captured on a laptop using SPSS Version 29 (SPSS Inc., Chicago, IL, USA) to make the data ready for analysis.

#### 2.4.5. Variables of Interest

The variables of interest for analysis in our study included:

1. Dependent variable: TB non-adherent patients.
2. Independent variables: Socio-demographic, program-related, and drug-related factors.

The primary outcome variable was the prevalence of non-adherence, assessed through patients’ self-reports on medication intake. Patients were asked if they had missed any doses; those who reported missing more than 10% of their prescribed medication (i.e., taking less than 90%) were classified as non-adherent.

A combination of statistical tests were used to analyze the data and identify potential predictors.

*Descriptive statistics*: These were used to summarize and describe the characteristics of the sample.

Examples include calculating the means, medians, standard deviations, percentages, and frequencies for the various variables.

*Pearson Chi-square test*: This was used to examine the association between categorical variables.

It was used to determine if there was a statistically significant relationship between, for example, demographic factors (like age and gender) and non-adherence rates, and the factors associated with non-adherence.

### 2.5. Ethical Considerations

The study was conducted according to the guidelines of the Declaration of Helsinki. The study proposal received approval from the Human Research Ethics Committee (HREC) of the Faculty of Medicine and Health Sciences at Walter Sisulu University. The ethics approval was issued on 16 August 2023 as indicated in the Ethics Approval Certificate Letter. The certificate letter registration number 047/2023 was issued.

## 3. Results

The results are presented in order reflecting the aims and objectives of the study.

### 3.1. Demographic Characteristics

The demographics determined were gender, age, marital state, educational background, income status, and HIV status. Of the 65 patients interviewed, 41 (63.08%) were males and 24 (36.92%) females. The mean age ± STD age of patients was 36.23 ± 11.21 years. The majority were in the age range 26–35 and 53 (81.54%) were single. The majority 53 (81.54%) had a high school level of education. There were 35 unemployed individuals (53.85%) and 35 (53.85%) patients had an HIV status of sero-negative (Table 1).

### 3.2. Demographics of Patients and Adherence Status

#### 3.2.1. Prevalence of Adherence and Non-Adherence Patients

There were 45 (69.23%) adherent patients and 20 (30.77%) non-adherents. Of these, 11 (55.00%) non-adherents were males and 9 (45.00%) were females. Among the 24 (36.92%) females, 15 (33.33%) were adherent and 9 (45.00%) were non-adherent. 

Gender had a major impact, with 11 (55.00%) of the males being non-adherent and 9 (45.00%) of the females. 

Majority 10 (50.00%) were non-adherents in age range of 26–35 years, followed by 4 (20.00%) in the age range 36–45 years, then 3 (15.00%) in both age ranges of 18–25 years and above 55 years. 

Non-adherence was observed amongst single people with a higher number of 15 (75.00%) followed by patients who were married with 5 (25.00%); hence, marital status had a big influence. 

Those with high school level of education had a greater number of 13 (65.00%) non-adherents than those with tertiary level of education accounting for 7 (35.00%).

Unemployed patients had the greatest number of non-adherents totaling to 13 (65.00%), followed by those earning ZAR 1000–3000 per month with 6 (30.00%) and 1 (5.00%) in those earning >5000 per month.

A higher number of Sero-positives were non-adherent with 11 (55.00%), and 9 (45.00%) of Sero-negative patients as reflected in Table 2. 

#### 3.2.2. Association Between Demographics and Non-Adherents

The association of sociodemographic characteristics with non-adherence to TB treatment was determined. Of the 65 patients, 45 (69.23%) adhered to therapy, whereas 20 (30.77%) did not. 

Gender had a major impact, with males reporting higher numbers of adherents of 30 (66.67%) and non-adherents of 15 (33.33%) than females with 11 (55.00%) adherents and 9 (45.00%) non-adherents. 

The age range of 26–35 years had higher numbers of adherents at 16 (35.56%) and 10 (50.00%) non-adherents.

With respect to marital status, single adherents accounted for 38 (84.44%) and 15 (75.00%) were non-adherents. 

Patients with high school level of education included 40 (88.89%) adherents, and 13 (65.00%) non-adherents, while patients with tertiary level of education included 5 (11.11%) adherents and 7 (35.00%) non-adherents. 

Unemployed patients included the greatest number of adherents at 22 (48.89%) and 13 (65.00%) non-adherents, followed by those earning ZAR 3001–5000 with 9 (20.00%) adherents and they were none non-adherents. Those earning >ZAR 5000, accounted for 8 (17.78%) and only 1 (5.00%) patient non-adhering. Then 6 (13.33%) adherents in the lowest income range of 1000–3000 and 6 (30.00%) non-adherents. 

In terms of HIV-Status, Sero-positive had a higher number of 11 (55.00%) while Sero-negative accounted for 9 (45.00%) as reflecting in Table 2. Sero-negative patients had a higher number of adherents of 26 (57.78%) and 9 (45.00%) non-adherents, compared to 19 (42.22%) sero-positive adherents and 11 (55.00%) non-adherents.

Gender was identified as a major predictor of adherence (Table 2). Males were more adherent to treatment than females with a significant association of (X^2^ = 65.00 and *p* < 0.001). Age, marital status, educational level, employment status and HIV status were all not statistically significant predictors of non-adherence (Table 2).

### 3.3. Factors Influencing TB Patients’ Noncompliance with Therapy

The factors influencing TB patients’ noncompliance with therapy were identified. Of these, distance from the clinic and a lack of support from the family had the largest effect, followed by unemployment, then substance abuse and adverse effects of treatment. Shortage of drugs at the clinic and poor interaction with staff had the smallest effect as demonstrated in Table 3. The interpretation and description of the specific outcomes within a broader research context are considered in the Discussion section in Section 4.

## 4. Discussion

The aim of this study was to assess the determinants of non-adherence in TB patients receiving TB treatment and attending a public primary health care setting. For effective TB control and eradication, treatment adherence is essential. The sample in this study included 63.08% males and 36.92% female participants, with the majority in age range of 26–35 years. The age range (26–35 years) in this study reflects evidence that more than 90% of global TB cases and deaths occur in the age group 15–54 years [34].

In the current study, 69.23% of the participants adhered to ATT, while 30.77% were non-adherent. These results indicate 6.3% lower adherence than that observed in a study carried out in northeast Ethiopia revealing adherence of 75.50% [35], and of 77.50% in a study in Ethiopia [36] and 79.20% in South Africa [34]. The disparity in these outcomes might be explained by differences in the definition of non-adherence to anti-TB medication. There is presently no agreement on the definition of adherence to ATT, despite the recommendations of the WHO on the quantity and timing of missed medications or hospital appointments [23].

In the current study, about 30.77% of the patients were non-adherent to ATT, which was 5.5% higher than observed in northeast Ethiopia [35]. This could be attributed to factors such as level of education, socio-economic status, HIV co-infection, and adverse effects and the waiting period at the health care facility, which is consistent with another study performed in South Africa [36]. This is further supported by the results from north-east Ethiopia [35]. This underscores the importance of health education and awareness campaigns to improve treatment adherence and outcomes as recommended in a study performed in southwest Nigeria with adherence of 77.50% [37].

Additionally, this may be because self-reports were used to quantify non-adherence, as patients may be prone to overestimate their adherence when using this approach [38]. An overestimation of 27.00% in the degree of adherence was suggested when self-reports were utilized in one study [39]. Nonetheless, it was pointed out that patients who acknowledge and disclose noncompliance readily embrace and execute treatments aimed at improving their everyday lives [40].

It has been observed that doctors typically do not correctly anticipate the adherence levels of their patients based on their knowledge of these patients, assuming that the degree of non-adherence is low [41].

Unemployment was also linked to non-adherence to anti-TB medication, particularly in low- and middle-income countries where TB is prevalent. Poverty can create barriers to accessing and completing TB treatment, impacting both the individual patient and public health efforts to control the disease [42]. In this study, a significant number of participants (65.00%) were non-adherent due to unemployment and receiving less than one thousand rand (<ZAR 1000) monthly.

Results from this study revealed that 55.00% of sero-positive patients compared to 45% of sero-negative patients were non-adherent. This is consistent with results obtained in a study in patients with TB/HIV co-infection [43]. It is possible that these patients will not follow their TB treatment plan. Patient-related factors like a heavy pill load, bad treatment responses, and anxiety over not being able to handle both anti-TB medication and ART could be the cause of this [14].

In a current study, 55.00% of participants reported the use of substance use as among the factors contributing to non-adherence. Substance use can lead to a cycle of relapses and recovery, making it challenging to attend appointments consistently and follow medication schedules [44].

Of the non-adherent patients, 35.00% stated they had stopped taking their medication due to a shortage of treatment at the clinics. Consequently, they would be referred elsewhere, such as to their closest public pharmacy, to be given their treatment or they would buy from private pharmacies. This issue can significantly impact patient care, treatment outcomes, and the overall functioning of the healthcare system [45].

Results from this study indicated that due to poverty, 65.00% of participants could not afford to buy the medicines or had no transport money to go to other public pharmacies. This leads to patients not completing their recommended dose, leading to a compromised immune system contributing to TB complications like MDR-TB, with potential further development to XDR-TB. Poor adherence to anti-TB medication is a major barrier to global control, being estimated to be as low as 40% [46]. Multiple factors that influence adherence to treatment include economic and structural factors, such as homelessness, unemployment and poverty, as well as patient-related factors like ethnicity, gender, age, knowledge about TB, cultural belief systems, and mental state. 

The results of this study revealed that 70.00% of participants reported that the distance from their home to the clinic for reviews or for renewing their monthly TB treatment was a hindrance to adherence. Longer distances mean more time spent traveling to and from the clinic for appointments, which can be a burden, especially for those with busy schedules or limited mobility [47].

A lack of family support was expressed by 70.00% of the participants, who stated that they had a poor relationship and poor communication with their family members. Family support, especially during the intensive phase of treatment, plays a crucial role in a patient’s ability to adhere to his or her medication regimen [48].

Adverse drug reactions (ADRs), or side-effects, are a significant contributing factor to non-adherence to TB treatment [49]. Patients experiencing unpleasant side-effects from TB medications may be less likely to adhere to their treatment regimen. This potentially leads to treatment failure, drug resistance, and increased morbidity and mortality. In the current study, 55.00% reported that they could not continue with their treatment after experiencing side-effects like nausea or diarrhea.

Finally, the least important factor, reported by 15.00% of the participants, was that poor interaction with the staff at the clinic contributed to non-adherence to TB treatment. The participants expressed their view that they were not given time and space to properly communicate their experience about the treatment, such as having adverse effects. This led them to have insufficient knowledge about the disease and why they should be taking the medication. Poor interactions with clinic staff can indeed contribute to medication non-adherence. When patients feel disrespected, misunderstood, or unsupported by healthcare providers, they are less likely to follow treatment plans. This can manifest as missed appointments, incorrect medication dosages, or even complete cessation of treatment [50].

### 4.1. Strengths and Limitations

#### 4.1.1. Strengths

The following are the strengths of the current study:

*Snapshot of prevalence:* As a cross-sectional study, it is well suited for capturing the prevalence of non-adherence and its associated factors within a population at a specific point in time.

*Efficiency and cost-effectiveness:* Such studies are often simpler and less expensive to conduct than longitudinal studies, making the current study useful as a preliminary investigation with limited resources.

*Identifying potential risk factors:* The study contributes to identifying potential risk factors associated with non-adherence, including socioeconomic factors, health system issues, and individual characteristics. 

#### 4.1.2. Limitations 

*Difficulty in establishing causality:* Since the data were collected at one point in time, it is difficult to determine whether a particular factor directly causes non-adherence or if there is a causal relationship between variables.

*Limited longitudinal tracking:* The study does not allow for tracking changes in adherence over time, making it hard to understand the dynamics of non-adherence behaviors.

*Potential for bias:* Self-reported data, common in cross-sectional studies including the current study, can be prone to recall bias or social desirability bias.

*Generalizability concerns:* The sample selected was not representative of the broader population, limiting the generalizability of the findings.

*Data collected in one week:* This was a limitation of the study given the variability in patient attendance and other operations of the healthcare facility.

### 4.2. Implications and Recommendations

#### 4.2.1. Implications

*Treatment failure:* Non-adherence can lead to treatment failure, continued infectiousness, and the development of drug-resistant TB.

*Public health risk:* Untreated or improperly treated TB poses a significant risk of transmission to others.

*Increased treatment costs:* Non-adherence can lead to increased healthcare costs due to readmissions, longer treatment durations, and the need for more expensive treatments.

#### 4.2.2. Recommendations

*Comprehensive counseling:* TB patients should be thoroughly counseled on the importance of adhering to treatment, potential side-effects, and the need for follow-up appointments.

*Improved health education:* Patients and their families should receive comprehensive education about TB, its transmission, and the importance of adherence to treatment.

*Stronger social support:* Strengthening social support networks through family involvement, community support groups, and peer support can significantly improve adherence.

*Addressing systemic barriers:* Addressing systemic barriers such as long waiting times, inadequate transportation, and a lack of access to healthcare facilities can improve adherence.

*Directly observed therapy (DOTS):* Implementing DOTS, where healthcare providers directly observe patients taking their medication, can significantly improve adherence.

*Further studies:* Further research should be carried out in more than one healthcare facility.

## 5. Conclusions

In conclusion, this study evaluated the determinants of non-adherence and the prevalence of non-adherence when taking anti-TB drugs. Gender was identified as a major variable impacting treatment adherence (Table 2). Age, marital status, educational level, employment status and HIV-Status, were all studied, but none were shown to be statistically significant predictors of non-adherence. Socioeconomic status, side-effects of drugs, HIV co-infection, and support from clinic staff and family were all factors linked to non-adherence to anti-TB medication. Furthermore, the study offers valuable insights into barriers to TB treatment adherence, but is limited by its small sample size, single-site design, short period of data collection, and reliance on self-reported data. Future research should include larger, multi-site samples and longitudinal designs to better understand the determinants of non-adherence and inform interventions. Comprehensive treatment programs should be promoted to address the multifactorial determinants of non-adherence to TB treatment.

## Figures and Tables

**Table 1:** Demographic characteristics of TB patients (*n* = 65).

Variables	Frequency (*n*)	Percentage (%)
**Gender**		
Male	41	63.08
Female	24	36.92
**Age range**		
18–25	13	20.00
26–35	26	40.00
36–45	14	21.54
46–55	4	6.15
>55	8	12.31
**Marital Status**		
Single	53	81.54
Married	12	18.46
**Educational Status**		
High School	53	81.54
Tertiary Level	12	18.46
**Income Status**		
Unemployed	35	53.85
1000–3000	12	18.46
3001–5000	9	13.85
>5000	9	13.85
**HIV Status**		
Sero-positive	30	46.15
Sero-negative	35	53.85

**Table 2:** Sociodemographic characteristics and their association with treatment non-adherence.

Sociodemographic Variables	Treatment Adherence and Non-Adherence		Pearson Chi-Square Tests	
	Adherents *n* %	Non-Adherents *n* %	X^2^	*p* Value
**Gender** *n* %			65	<0.001
Male 41 (63.08)	30 (66.67)	11 (55.00)		
Female 24 (36.92)	15 (33.33)	9 (45.00)		
**Age range**			3.394	0.494
18–25 13 (20.00)	10 (22.22)	3 (15.00)		
26–35 26 (40.00)	16 (35.56)	10 (50.00)		
36–45 14 (21.54)	10 (22.22)	4 (20.00)		
46–55 4 (6.15)	4 (8.89)	0 (0.00)		
>55 8 (12.31)	5 (11.11)	3 (15.00)		
**Marital Status**			0.142	0.706
Single 53 (81.54)	38 (84.44)	15 (75.00)		
Married 12 (18.46)	7 (15.56)	5 (25.00)		
**Educational Status**			0.142	0.706
High School 53 (81.54)	40 (88.89)	13 (65.00)		
Tertiary Level 12 (18.46)	5 (11.11)	7 (35.00)		
**Income Status**			4.035	0.258
Unemployed 35 (53.85)	22 (48.89)	13 (65.00)		
1000–3000 12 (18.46)	6 (13.33)	6 (30.00)		
3001–5000 9 (13.85)	9 (20.00)	0 (0.00)		
>5000 9 (13.85)	8 (17.78)	1 (5.00)		
**HIV Status**			0.002	0.968
Sero-positive 30 (46.15)	19 (42.22)	11 (55.00)		
Sero-negative 35 (53.85)	26 (57.78)	9 (45.00)		

**Table 3:** Non-adherent patients due to patient program and drug-related factors.

Factors Affecting the Patient	Prevalence (*n*)	Percentage (%)
Substance use	11	55
Distance to the clinic	14	70
Adverse effects of the treatment	11	55
Poor interaction with the staff at the clinic	3	15
Lack of family support	14	70
Unemployment/Poverty	13	65
Shortage of drugs at the clinic	7	35

## Data Availability

Because data contain information that might jeopardize research participants’ privacy, the data supporting the study’s conclusions are not publicly available.

## Abbreviations

The following abbreviations are used in this manuscript:

WHO	World Health Organization
COVID	Corona Virus Disease
HIV/AIDS	Human Immunodeficiency Virus/Acquired Immunodeficiency Syndrome
UN	United Nations
TB	Tuberculosis
ART	Antiretroviral Treatment
SA	South Africa
MDG	Millenium Development Goals
SDGs	Sustainable Development Goals
DOT	Directly Observed Treatment
EC	Eastern Cape
ATT	Anti-Tuberculosis Treatment
ADRs	Adverse Drug Reactions
SIAPS	Systems for Improved Access to Pharmaceuticala and Services
MDR-TB	Multidrug-Resistant Tuberculosis
XDR-TB	Extreme Drug-Resistant Tuberculosis
